# THE EFFECTS OF IMMIGRANT STATUS ON WELL-BEING AMONG OLDER ADULTS BY RACE-ETHNICITY: A MULTI-GROUP ANALYSIS

**DOI:** 10.1093/geroni/igz038.2621

**Published:** 2019-11-08

**Authors:** Heehyul Moon, Hyesook Kim, Sunshine Rote, William E Haley

**Affiliations:** 1 University of Louisville, Louisville, Kentucky, United States; 2 Daegu University, Daegu, South Korea, Korea, Republic of; 3 University of South Florida, Tampa, Florida, United States

## Abstract

Although prior researchers have decried the lack of research on racial/ethnic minority older adults, they have been less vocal about the gaps in research concerning the ways in which immigrant status and race/ethnicity affect their well-being. Thus, we examined the role of immigrant status on the stress coping process by race/ethnicity using the Transactional Model of Stress and Coping. The multi-group analysis function in structural equation modeling was used to determine whether the stress coping process was equivalent across three racial/ethnic groups (Non-Hispanic White (NHW), Non-Hispanic Black(NHB), and Hispanic) by immigrant status using the Round 1 of the National Health and Aging Trends Study (NHATS, (U.S.-born= 4,799, foreign-born=612)). We found that immigrant status and race/ethnicity may have complex effects on the stress coping process. For example, the total effects of being an immigrant were significantly associated with more stressors, less resources, and worse physical health. Except NHW, the total effects of being immigrant were associated with higher levels of depression and anxiety. With respect to the direct and indirect effect of immigrant status in the three groups, the Hispanic group has a larger effect of immigrant status on stressors, resources, depression/anxiety and physical health than their NHW and NHB counterparts. The results indicated that immigrant racial/ethnic minority older adults were more likely to have higher levels of depression and anxiety than the U.S.-born except for NHW. Immigrant status will require special attention in both assessment and management of depression/anxiety among racial/ethnicity minority older adults.

